# Experimental study on operation limit of ground heat exchanger based on ground source heat pump unit

**DOI:** 10.1371/journal.pone.0319430

**Published:** 2025-03-11

**Authors:** Rui Zhang

**Affiliations:** Shandong Institute of Petroleum and Chemical Technology, Donging, Shandong, China; University of Shanghai for Science and Technology, CHINA

## Abstract

The heat transfer performance of a ground heat exchanger (GHE) directly influences the operational performance of a ground source heat pump (GSHP) system. The fluid temperature within the GHE is constrained by the protective temperature limits of the GSHP unit. Specifically, the inlet water temperature has an upper limit in summer and a lower limit in winter. These temperature limits further affect the heat exchange efficiency between the GHE and the surrounding soil. In this study, an experimental station featuring a single U-shaped GSHP system was constructed, and a three-dimensional model of the system was developed. Experiments were conducted by operating one or two GHEs to investigate the heat transfer per unit well depth and the matching relationship between cooling capacity and indoor load when the inlet water temperature of the heat pump unit approaches its summer and winter limits. In summer, when operating a single GHE, the heat transfer per unit well depth reached 134.4 W/m at an inlet temperature of 45 °C. When the cooling supply just matched the cooling load demand, the heat transfer per unit well depth was 131.5 W/m. However, prolonged operation led to a scenario where the cooling supply could no longer meet the load demand. In winter, operating a single GHE resulted in a heat transfer per unit well depth of 43.95 W/m at an inlet temperature of 5 °C. These results indicate that when the number of heat exchangers is insufficient, the inlet water temperature of the heat pump unit may reach or exceed the limit value, leading to decreased unit efficiency. Additionally, inadequate heat exchange between the GHE and the soil results in insufficient cooling or heating capacity, failing to meet the indoor load requirements.

## 1 Introduction

The ground source heat pump (GSHP) system offers significant energy savings and environmental benefits, making it a popular choice in HVAC. A key factor affecting the system’s operation is the heat transfer performance between the ground heat exchanger (GHE) and the soil. This performance is influenced by the temperature difference between the fluid in the buried pipe and the surrounding soil. The GHE is constrained by the protection temperature of the GSHP unit. This results in a maximum inlet water temperature in summer and a minimum in winter. During periods of high indoor load demand, extended operation of the GHE, or insufficient GHE units, the inlet water temperature may become excessively high in summer or too low in winter. When the inlet water temperature exceeds its limits, the GSHP system cannot function properly.

Numerous studies have been conducted on GSHP systems. Jingyang Han et al. monitored the thermal performance of GSHP systems in large buildings and analyzed the impact of GHE spacing on economic efficiency. Their findings indicate that economic efficiency decreases as the spacing of the GHE increases [1]. Weeratunge H et al. examined the life-cycle economics of hybrid solar-assisted ground source heat pump (SAGSHP) systems, evaluating their economics and optimal sizing in heating-favored climates across Australia. Their results show that the life cycle costs of SAGSHP systems are lowest in areas with high or moderate solar irradiation, high electricity prices, and high or moderate natural gas prices [2]. Zhou K et al. developed a detailed numerical model to assess the thermal disturbance of the environment on ground thermal conductivity (λs) during thermal response tests (TRTs). Their research provides valuable insights for the effective implementation of TRTs [3]. Zhao Z et al. analyzed the probabilistic uncertainties of design variables in GSHP systems using reliability-based design optimization (RBDO). They assigned various uncertainty levels to three design variables—borehole depth, ground pipe radius, and mass flow rate—as well as to groundwater velocity and ground thermal conductivity. Their results highlight that variable uncertainty impacts system reliability and total cost determination [4]. Nguyen A employed an iterative spectral approach to determine the maximum heat pump capacity of hybrid GSHP systems, ensuring the longevity of specified ground exchanger fields. This approach accommodates various building load profiles and heat pump controls. Their findings suggest that maximum capacity is contingent upon actual conditions, emphasizing the importance of long-term simulations [5]. Meng X et al. investigated how groundwater seepage velocity and soil freezing affect GSHP performance. They established a full-scale dynamic simulation platform to quantitatively analyze these effects under typical geological conditions. This platform allows for coupling inlet and outlet temperatures of buried pipes with building loads, closely mirroring actual operating conditions [6]. Hu Z et al. proposed a heat source tower (HST) to mitigate soil thermal imbalances in GSHP systems. The HST absorbs heat from air and redistributes it to the soil via borehole heat exchange (BHE) during summer. They performed finite element iterative calculations using MATLAB software [7]. Zhang M et al. built a GSHP model for a building in Changsha using TRNSYS software. They optimized borehole depth, spacing, pipe length, and configuration based on a comprehensive annual coefficient of performance (COP all) of the heating system [8]. Gao B et al. identified common operational issues in GSHP systems by assessing 26 installations across different regions under typical operating conditions. Their study advocates for the use of GSHP systems in western China [9]. Hao LA et al. examined the impacts of groundwater advection, soil thermal properties, the arrangement of ultra-long flexible heat pipes, and operating conditions on ground temperature variations in GSHP systems using computational fluid dynamics [10]. Zhang H developed a 3D finite element dynamic simulation platform for GSHP systems, analyzing simultaneous freezing flow and groundwater effects in office buildings. Their results indicate that increasing hole spacing improves GSHP performance. They also determined differing soil freezing penetration distances across various regions [11]. Abdel-Salam M. R. H. et al. evaluated the heating performance of three vertical closed-loop water-to-air GSHP systems in individual homes in Innisfil, Canada, for one heating season. Their findings suggest that appropriate design and installation ensure system efficiency in cold climates [12]. Zhang H et al. studied the effects of pipe spacing on soil moisture radius, freezing time, freezing distance, and the unit average COP using a dynamic simulation platform that incorporates heat and moisture transfer, seepage, and freezing effects [13]. Sedaghat A et al. compared the long-term performance and initial costs of GSHP and air source heat pump systems in hot regions through numerical simulations. They also analyzed how variation in GHE length, pipe spacing, depth, and diameter affect system performance [14]. Roohidehkordi I. et al. simulated the long-term impacts on subsurface temperature of GSHP systems, revealing that geothermal heating boosts biodegradation [15]. Dicarlo A. explored how soil permeability, irrigation pipe positioning, and soil water content influence the efficiency of GSHP systems in northeastern United States across seasons using computational fluid dynamics [16]. Shukla S. et al. designed a geothermal heat exchanger system using phase change materials (PCM), noting that PCM enhances thermal performance while reducing energy consumption and greenhouse gas emissions [17]. For hybrid GSHPs, Lee M et al. investigated how changes to the entering secondary fluid temperature (EFT), flow rate, and ground temperature impact heating performance. Their results stem from experimental studies [18]. Alshehri F. et al. focused on the design of GSHP systems in Saudi Arabia and similar arid regions. Their rigorous sensitivity analysis highlighted four critical design parameters: thermal conductivity, soil temperature, building load, and fluid flow rate [19]. Zhang Hongzhi et al. constructed a 3D simulation platform for a GSHP system in an office building, evaluating the influence of pipe arrangement and GHE type on system performance. They found that moisture transfer, seepage, and freezing contribute positively to heating COP, while only seepage benefits cooling COP [20]. Ma Z. et al. reviewed optimal design and control strategies for GSHP systems, utilizing sensitivity analysis to determine key variables. Their findings demonstrate that optimized control methods outperform rule-based approaches [21]. Xu, X. et al. used linear regression, nonlinear regression, and artificial neural networks to evaluate the heat transfer rate of ground source heat pumps. Their comparison revealed that artificial neural networks predict the heat transfer rate of ground source heat pumps more accurately than linear and nonlinear regression. The study analyzed the effects of well diameter, U-tube thickness, soil thermal conductivity, vertical well depth, flow velocity, and water temperature difference on the heat transfer rate of ground source heat pumps [22]. Zhou, K. et al. developed three-dimensional models of horizontal ground heat exchangers(HGHE), including spiral, slinky, and linear types, and examined the effects of various ground surface boundary conditions (BCs)—such as constant temperature, time-varying temperature, and energy balance BCs—on the short-term performance of these heat exchangers. The results indicated that the surface boundary conditions were more sensitive to linear HGHE than to spiral and slinky HGHE [23]. Hou G. et al. utilized TRNSYS software to simulate the overall performance of a hybrid GSHP system, analyzing energy variations and soil thermal conditions under contrasting heating/cooling settings [24]. You T. et al. established a GSHP system using energy piles, studying its heating performance in hotels located in Harbin, Changchun, Shenyang, and Beijing [25]. Luo Y. et al. created a GSHP model based on deep borehole heat exchangers with non-uniform pipe insulation. They conducted numerical calculations using a segmented finite line-source method and validated their model with annual field test data [26]. Hou G. et al. simulated a hybrid GSHP system based on a liquid dry cooler (LDC) using TRNSYS software, finding the hybrid system outperformed traditional systems in soil heat storage and overall performance [27]. Warner J. developed a one-dimensional Underground Thermal Battery (UTB) model, which is shallower and less expensive to install than standard vertical borehole GHEs. The simulation results from this one-dimensional model were corroborated with actual data and three-dimensional UTB model simulations, enhancing computational efficiency and annual performance predictions [28]. Chen Y. et al. integrated a SAGSHP system for a150 m^2^ rural house near Shanghai, implementing four practical heating modes. Their results indicate that a system with an installed collector operates more efficiently, reducing 79.6% of the AC demand during peak evening hours [29]. Georgiev A. et al. created a hybrid installation that includes a solar collector and GSHP unit capable of five operational modes. They proposed a methodology for assessing different system energy efficiencies and obtained performance data across various operational modes. Their results confirmed the superior performance of GSHPs for heating applications [30].

At present, existing research primarily focuses on optimizing the heat exchange performance of GHEs, addressing soil heat balance issues, and enhancing both cooling and heating performances of GSHP systems, including hybrid configurations. However, studies often fail to consider the simultaneous operation of GSHP units and GHEs. For instance, when the inlet water temperature limit of the heat pump is reached, the impact on the heat transfer performance of both GHEs and the heat pump is overlooked. In practical scenarios, particularly during summer, prolonged operation of GHEs can result in elevated soil temperatures. This creates a reduced temperature difference between the circulating fluid and the soil, leading to inadequate heat exchange and higher outlet temperatures for the GHEs. Consequently, this can increase the condensing temperature of the heat pump, decrease its operational efficiency, and impair cooling supply. If the outlet temperature exceeds the heat pump’s limit, it may even lead to a shutdown of the unit. Thus, it is crucial to assess the heat transfer performance of the GHE in conjunction with the performance of the heat pump unit. This research provides valuable guidance for similar projects.

This paper builds on experiments conducted with GSHP systems. It investigates the heat exchange per unit well depth of GHEs when the inlet water temperature at the heat pump unit reaches its threshold. It also explores the relationship between cooling capacity and indoor load. A three-dimensional model of a single U-shaped vertical GHE is created, and simulated values are compared with experimental data..

## 2 Experiment methods

### 2.1 Experimental system devices

**Fig 1:**
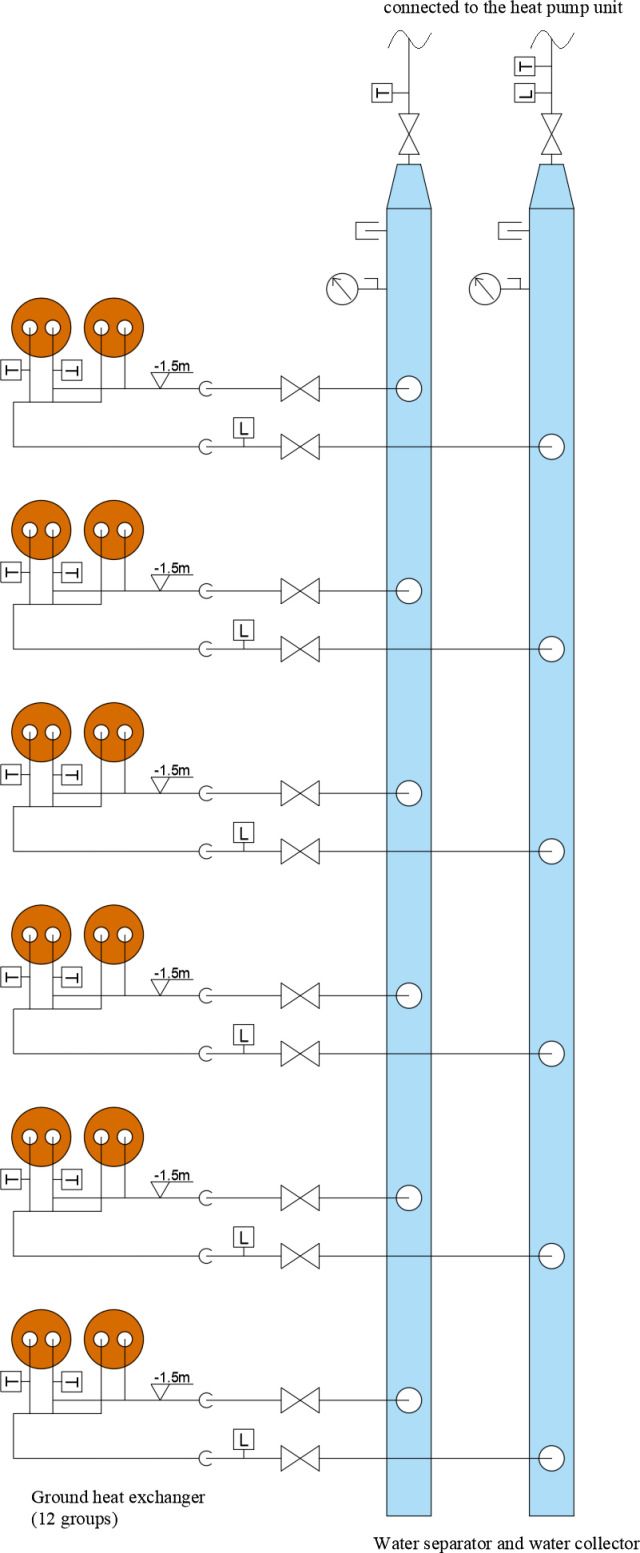
GHE system.

**Fig 2:**
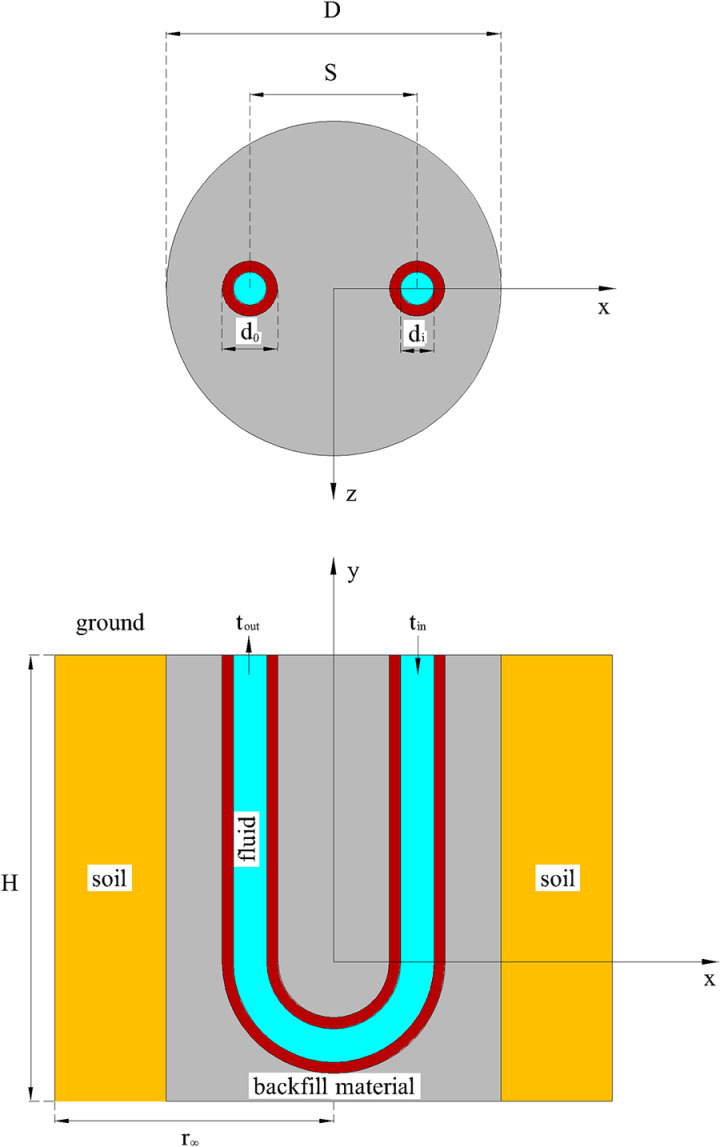
Vertical single U-shaped buried pipe heat exchanger.

The experimental system is based in Shanghai and utilizes a GSHP to provide cooling in summer and heating during winter for two offices, each measuring 94.5 m^2^. The system consists of a heat pump unit, circulating water pump, GHEs, water separator, water collector, expansion tank, indoor air conditioning terminal, and a pipeline system. The heat pump unit has a rated cooling capacity of 28 kW and a power consumption of 4.25 kW. Its rated heating capacity is 31.5 kW, with a power consumption of 4.09 kW, using refrigerant R410A. The circulating water pump for the GHE has a rated flow of 9 m^3^/h and a rated pump head of 25 m. Fig 1 illustrates the GHE system, which includes 12 wells numbered from 1 to 12. The GHE features a vertical single U-shaped tube, detailed in Fig 2, and the geometrical dimensions of the heat exchanger are listed in Table 1.

**Table 1:** Geometrical dimensions of GHE.

Parameter	Value(mm)	Parameter	Value(mm)
Inside diameter of U-shaped pipe *d*_i_	25	The depth of the borehole H	80000
Outside diameter of U-shaped pipe *d*_o_	32	The diameter of the borehole D	130
The center distance between the two branches of the U-shaped pipe S	65	The radius of the far boundary *r*_∞_	4000

### 2.2 Experimental data acquisition system

#### 2.2.1 Test of soil and circulating fluid temperature.

The temperature range for testing is between 0 and 60 °C. A PT100 thermal resistance sensor measures the temperature of the soil and the circulating liquid in the GHE. The opposite end of the sensor connects to the ADAM4015 data acquisition module. To ensure accuracy, a water bath calibration is conducted before setting up the thermal resistance. The water bath temperature is measured using a mercury thermometer, which has a range of 0–100 °C and an accuracy of ±0.1 °C. After applying the calibration correction, temperature measurement values are re-verified to meet accuracy standards.

**Fig 3:**
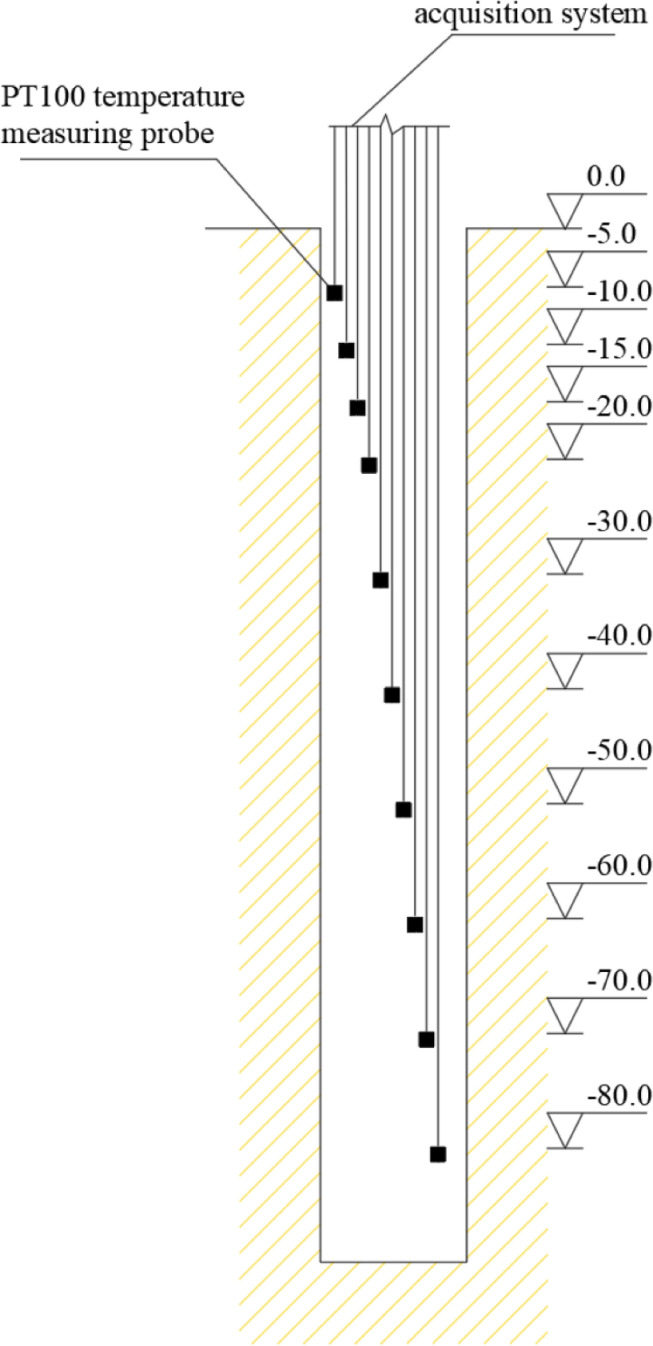
Layout of ground temperature measuring points.

**Fig 4:**
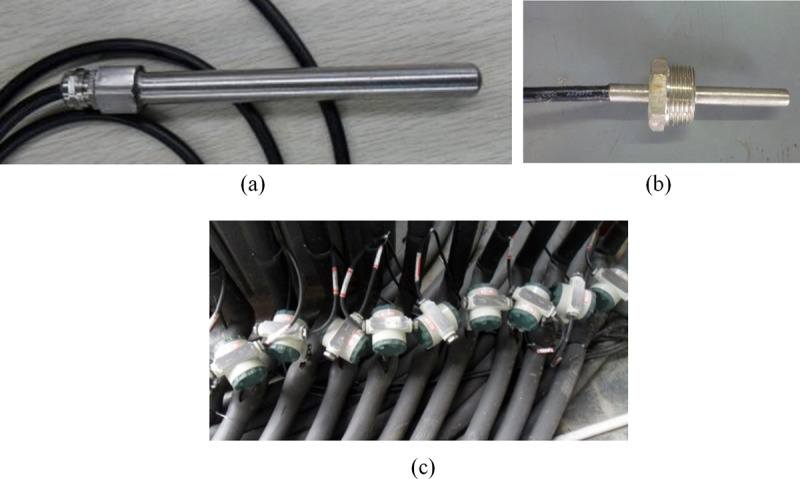
Measuring instrument (a) Armored thermal resistance for high voltage and waterproof ground (b) Thermal resistance of measuring the inlet and outlet water temperature (c) Turbine flowmeter of testing GHE flow.

The PT100 thermal resistance sensors are installed on the pipe walls in wells 11 and 12 to monitor the soil temperature surrounding the GHE. The sensors are buried at depths of 5m, 10m, 15m, 20m, 30m, 40m, 50m, 60m, 70m, and 80m. The layout of the ground temperature measuring points is shown in Fig 3. Fig 4(a) displays the armored thermal resistance, which is suitable for high pressure and waterproof temperature monitoring. The stainless steel protective sleeve has a diameter of 16mm, a length of 15 cm, and can withstand a maximum pressure of 1.5MPa.

Each GHE is equipped with PT100 thermal resistance sensors at the inlet and outlet to measure water temperature. The threaded thermal resistance for this purpose is shown in Fig 4(b).

#### 2.2.2 Indoor and outdoor temperature measurement.

Indoor and outdoor temperature and humidity are measured using self-evaluating temperature and humidity sensors. The temperature measurement range is from −20 °C to 60 °C with an accuracy of ±1 °C. The relative humidity measurement range is 0 to 95% with an accuracy of ±1.5% (at 60% RH and 25 °C). Outdoor measuring points are placed away from significant cold or heat sources and are shielded from direct sunlight. Indoor measuring points are positioned at various locations, 1.5 m above the ground.

#### 2.2.3 Flow rate measurement.

Each GHE includes a turbine flowmeter to measure the flow rate. The pipe diameter is DN10, with a measurement range of 0.2 to 1.2 m^3^/h and a working pressure of 6.3 MPa or less. Fig 4(c) shows the turbine flowmeter installation.

#### 2.2.4 Summary of measuring parameters and measuring instruments.

Table 2 summarizes the measuring parameters and instruments required for the experiment.

**Table 2:** Measuring parameters and measuring instruments.

Parameter		Measuring instrument
The temperature of the soil (The well wall temperature)	$t_{b}$	PT100 thermal resistance (Armored thermal resistance for high pressure and waterproof ground temperature monitoring)
The inlet water temperature	$t_{in}$	PT100 thermal resistance
The outlet water temperature	$t_{out}$	PT100 thermal resistance
Indoor temperature	$t_{n}$	Temperature self-evaluation
Outdoor temperature	$t_{w}$	Temperature self-evaluation
The flow rate	*V*	Turbine flowmeter

### 2.3 Experimental process and conditions

The GSHP system provides cooling in summer and heating in winter for the office. The number of buried pipes in operation is adjusted based on indoor load. To approach the protective temperature of the heat pump unit, the experiment is conducted with only one or two buried pipes running. The protection temperature limits are set to 45 °C in summer and 10 °C in winter. Prior to the experiment, the GSHP system is stopped for one day to allow the ground temperature to stabilize. During the experiment, one or two buried pipes are activated, followed by the pump, the heat pump unit, and finally the indoor unit, with indoor temperatures set to 26 °C in summer and 22 °C in winter. The data acquisition system collects parameters from Table 1 and connects them to the computer for analysis. Experimental conditions are outlined in Table 3.

**Table 3:** Experimental condition.

Season	Operating condition	Operating time
Summer	Operate one buried pipe11#	July 14, 10:26 to 19:00
	Operate two buried pipes 1# and 3#	July 22, 9:20 to 13:40
Winter	Operate one buried pipe12#	January 5, 19:30 to 22:00
	Operate two buried pipes11# and 12#	January 5 from 9:00 to 12:20

### 2.4 Data reduction

The heat exchange of ground heat exchanger is calculated as formula (1) [31].


$$Q=c_{p}\rho V(t_{in}-t_{out})$$ (1)

Where, *Q* is total heat exchange of ground heat exchange, W; *V* is volume flow of circulating liquid, m^3^/s; $t_{in}$ is water inlet temperature of ground heat exchange, °C; $c_{p}$ is specific heat of circulating liquid, J/(kg°C); $t_{out}$ is outlet temperature of ground heat exchange, °C; *ρ* is density of circulating liquid, kg/m^3^.

The calculation of heat exchange per unit well depth is shown in formula (2) [31].


$$q=\frac{Q}{H}$$ (2)

Where, *q* is heat exchange per unit well depth, W/m; *H* is buried pipe well depth, m.

Define the overall heat transfer coefficient refer to [32] and [33] as the follow.

The heat flux can be calculated as equation(3).


$$q^{\prime }=\frac{Q}{\pi dl}$$ (3)

Where, *q’* is heat flux, W/m^2^; *d* is the inside diameter of the U-shaped pipe, m; *l* is the long of the U-shaped pipe, m.

We define the overall heat transfer coefficient between the borehole wall and the fluid as the equation (4).


$$K=\frac{q^{\prime }}{t_{b}-t_{f}}$$ (4)

Where, K is the heat transfer coefficient, W/(m^2^ °C); *t*_*b*_ is the well wall temperature, °C; *t*_*f*_ is the temperature of the fluid, °C.

### 2.5 Experimental uncertainty analysis

Experimental uncertainty may arise from the instruments, environment, and readings. An uncertainty analysis is essential to validate the accuracy of the experiment. In this study, the uncertainties of both measurement and calculation parameters are assessed and analyzed.

The measurement parameters include temperature and flow rate. The uncertainty associated with these parameters is calculated using equation (5). The relative uncertainty of the measurement parameters is determined through formula (6). The primary calculation parameter is the heat transfer of the GHE. The relative uncertainty of the calculated parameters is obtained using formulas (7) and (8) [34].


$$\delta x_{i}=A\cdot \gamma_{i}$$ (5)

$$\delta Rx_{i}=\frac{\delta x_{i}}{x_{i}}$$ (6)

$$F=f(x_{i})�i=1\cdots n$$ (7)

$$\delta RF=\frac{\sqrt{\sum_{1}^{n}{\left(\frac{\partial F}{x_{i}}\delta x_{i}\right)}^{2}}}{F}$$ (8)

Where, *x_i_* is measurement parameters, *δx_i_* is the relative uncertainty of the measurement parameters, *A* is the upper limit of the measuring range, and *γi* is the accuracy grade according to the manufacture. *δRx_i_* is the relative uncertainty of the measurement parameters, *δRF* is the relative uncertainty of the calculated parameters, *F* is the calculated parameters, *f*(*x_i_*) is the function of the measurement parameter *x_i_*.

The working condition of the GHE on July 14 during the summer was selected as a typical scenario. The relative uncertainty of each parameter’s typical values was calculated, with results presented in Table 4. It shows that the relative uncertainty for both measurement and calculation parameters is below 5%. Thus, the experimental results are deemed accurate and reliable.

**Table 4:** Relative uncertainties of the main parameters in the experiment.

Parameter	Data type	Typical value	Relative Uncertainty
Average well wall temperature	measurement parameter	27.46 °C	0.36%
Average inlet temperature of GHE	measurement parameter	34.22 °C	0.29%
Average outlet temperature of GHE	measurement parameter	27.22 °C	0.37%
Average outdoor temperature	measurement parameter	30.6 °C	3.3%
Average indoor temperature	measurement parameter	28.1 °C	3.6%
Average flow rate	measurement parameter	0.375 m^3^/h	2.7%
The heat transfer of the ground heat exchanger	calculation parameter	3062.5W	3.3%

## 3 Simulation of GHE

### 3.1 Physical model

In a standard single U-shaped vertical buried pipe heat exchanger, a U-shaped pipe is installed in a drilled well. The well is sealed with backfill material to integrate it with the surrounding soil, as illustrated in Fig 2. During the operation of the buried pipe heat exchanger system, the U-shaped pipe is divided into inlet and outlet sections based on the fluid’s flow direction. The fluid enters the U-shaped pipe, flows to the bottom of the borehole, and then exits through the outlet section to facilitate heat exchange between the fluid in the pipe and the surrounding soil.

### 3.2 Condition setting

The geometric parameter settings are detailed in Table 1. The mathematical model, initial conditions, boundary conditions, grid division, and grid independence verification of the single U-shaped vertical buried pipe heat exchanger are referenced from [35]. Calculations were performed using numerical simulation software. The subsequent section will compare experimental results with simulation results.

## 4 Results and discussion

To align the inlet water temperature of the GSHP unit with its protection temperature, only one or two GHEs are operated during the experiment. This section analyzes the heat exchange per unit well depth of the GHE. It also examines the relationship between cooling capacity and indoor load when either one or two GHEs are operational, with the unit’s inlet temperature reaching its limit. In the experimental system, the inlet water temperature limit is 45 °C in summer and 10 °C in winter.

### 4.1 Summer condition

#### 4.1.1 One GHE runs separately.

##### (1) Experimental study:

**Fig 5:**
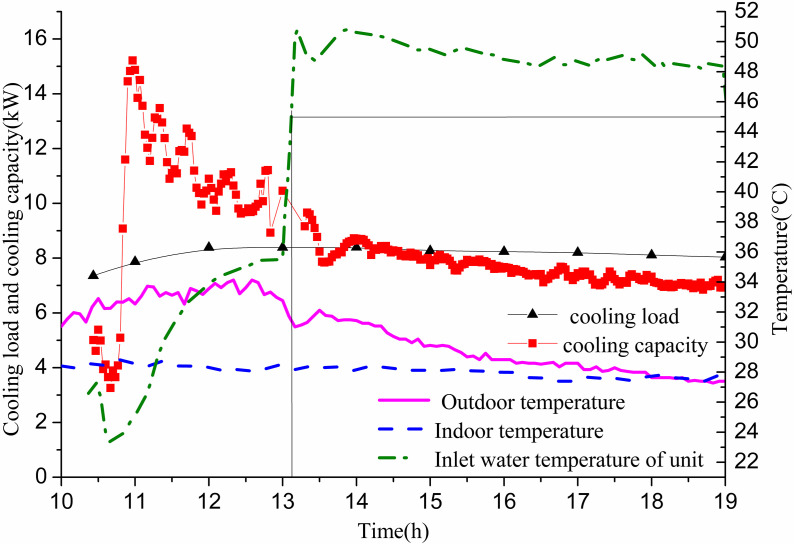
Variations of cooling capacity and indoor cooling load with operation time of single GHE operating in summer.

The experiment involves activating only the 11# GHE, operational from 10:26 to 19:00 on July 14, with a constant flow rate of 0.35 m/s. Fig 5 illustrates the relationship between indoor cooling load and cooling capacity. Initially, the cooling capacity falls short of meeting the indoor cooling load due to a relatively low inlet temperature from the GHE. The small temperature difference between the water and the soil results in insufficient heat extraction. To address this, the water temperature from the GSHP unit entering the GHE is increased. After approximately 0.5 h, the cooling capacity meets the indoor cooling load demand. This interaction causes the return water temperature of the heat exchanger—and thus the inlet water temperature of the GSHP unit—to initially be low, then rise rapidly, stabilizing around 50 °C. The time required for the unit’s inlet water temperature to increase from 26.6 °C to 45 °C is approximately 2.67 h (2 h and 40 min). At this stage, the cooling capacity still satisfies the indoor cooling load. After about 30 min, the cooling capacity equals the cooling load, marking a critical point. Subsequently, the cooling capacity fails to meet the indoor cooling load demand. The indoor temperature stabilizes around 28 °C, which is above the desired design temperature of 26 °C. At this point, the unit’s water inlet temperature reaches approximately 50 °C, resulting in significantly reduced unit efficiency.

**Fig 6:**
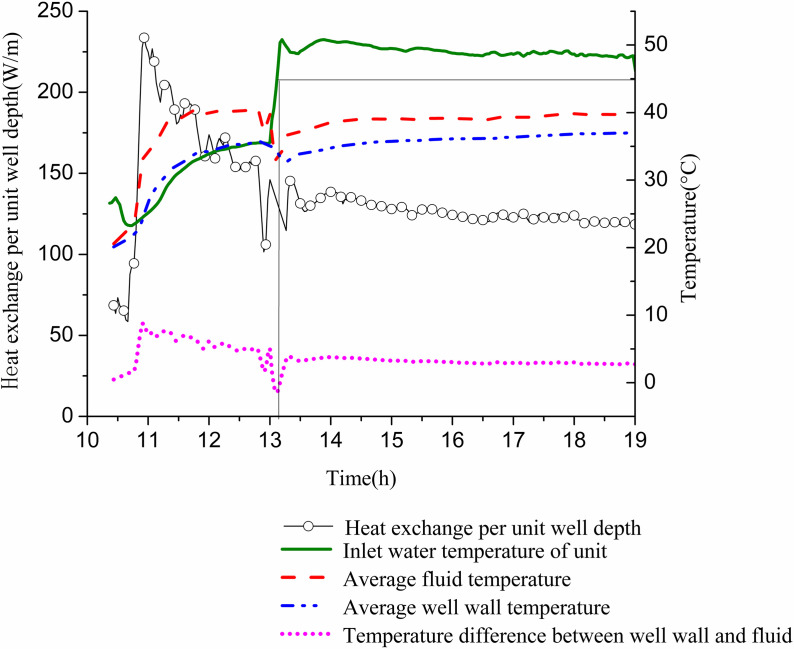
Variations of heat exchange per unit well depth and temperature difference between pipe wall and fluid with operation time.

Fig 6 illustrates the change in ground heat exchange per unit well depth and the temperature difference between the well wall and the fluid over time. The heat exchange per unit well depth increases rapidly during the first 40 min, followed by a gradual decrease with fluctuations. When the water inlet temperature reaches 45 °C, the heat exchange per unit well depth for the GHE is recorded at 134.4 W/m. At the point when the cooling capacity matches the indoor cooling load demand, the heat exchange per unit well depth measures 131.5 W/m. Subsequently, it continues to decline. By 19:00, the heat exchange per unit well depth drops to 118.47 W/m, representing 90.1% of the critical point heat exchange, while the cooling capacity fulfills 86.9% of the cooling load.

The temperature difference between the fluid and the well wall also rises sharply during the initial 40 min before gradually decreasing. When the unit’s inlet water temperature reaches 45 °C, the inlet and outlet water temperatures of the GHE are 47.17 °C and 31.16 °C, respectively, resulting in an average fluid temperature of 39.17 °C. The well wall temperatures at depths of 10 m, 15 m, 20 m, 30 m, 40 m, 50 m, 60 m, and 80 m are 37.12 °C, 37.14 °C, 36.13 °C, 37.14 °C, 34.95 °C, 33.31 °C, 31.69 °C, and 32.48 °C, with an average of 35 °C. The average temperature difference between the well wall and the fluid is measured at 4.17 °C. When the cooling capacity equals the cooling load demand, the buried pipes have inlet and outlet water temperatures of 44.99 °C and 29.43 °C, respectively. The average fluid temperature at this point is 37.21 °C, while the average well wall temperature is 33.9 °C, yielding a temperature difference of 3.31 °C. At the end of operation, the inlet and outlet temperatures are 46.18 °C and 33.34 °C, respectively, leading to an average fluid temperature of 39.76 °C and an average well wall temperature of 37.03 °C, with a temperature difference of 2.73 °C.

This data indicates that when the number of GHEs is inadequate, the inlet temperature of the heat pump unit exceeds the limit after prolonged operation. This results in a decrease in the refrigeration efficiency of the unit during summer, a reduction in heat exchange per unit well depth, and an inability to meet indoor cooling load demands.

##### (2) Comparison between theoretical calculation and experimental results:

**Fig 7:**
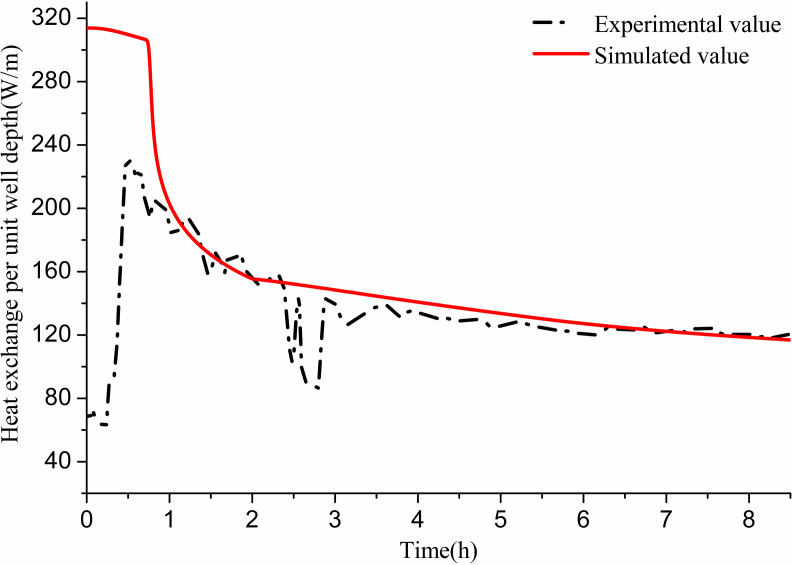
Comparison between experimental and simulated values of heat transfer per unit well depth of 11# GHE.

In the experiment, the GHE operated for approximately 9 h and then recovered for 13 h. Fig 7 presents a comparison between the simulated and experimental values of heat exchange per unit well depth over the 9-h operation period. During this time, the flow rate was 0.35 m/s, and the average inlet water temperature was 45 °C. The simulation results indicate that the heat exchange per unit well depth gradually decreases over time. In practical operations, while the heat exchange fluctuates with indoor load, it generally demonstrates a declining trend before becoming stable. The average heat exchange per unit well depth during the 9 h experimental operation was found to be 134.5 W/m. The simulation showed instability in the first 2 h. However, after this period, the average heat exchange per unit well depth was 130 W/m, resulting in a 3.3% error compared to the experimental findings.

**Fig 8:**
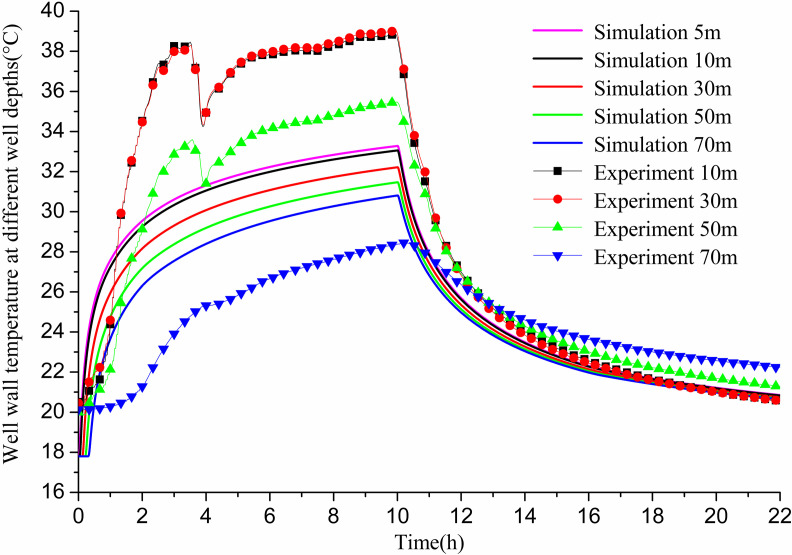
Comparison between experimental and simulated values of well wall temperature at different depths of 11# GHE.

Fig 8 compares the experimental and simulated values of well wall temperature at various depths of the GHE. Both the experimental and simulated results show a continuous increase in well wall temperature during operation, followed by a gradual recovery once operation ceases. After the recovery phase, at a depth of 10 m, the simulated value was 20.54 °C, while the experimental value was 20.2 °C, yielding a 1.7% error. At 30 m depth, the simulated value was 20.47 °C, and the experimental value was 20.3 °C, with an error of 0.83%. For 50 m depth, the simulated value registered at 20.41 °C, against an experimental value of 21 °C, presenting a 2.8% error. At a depth of 70 m, the simulated value was noted as 20.38 °C, with the experimental measurement at 22 °C, culminating in a 7.3% error. All errors remained below 10%, which is acceptable within the context of these measurements.

##### (3) Comparison with existing literature results:

Miyara A et al. [36] conducted an experimental analysis on the heat exchange performance of double-tube, U-tube, and multi-tube GHEs. They obtained the temperature distribution of the borehole wall and analyzed the heat exchange rate of different GHEs. The literature provides data on the variations in inlet and outlet water temperatures and heat exchange rates over time. While the test site referenced was Saga University in Japan, this study was conducted in Shanghai, China. Due to differing climates, the quantitative results vary; however, the trends in inlet and outlet water temperatures and heat exchange rates with operating time remain consistent.

In summer, the inlet and outlet water temperatures of the GHE rise quickly at first, then stabilize and increase slowly. Initially, heat transfer increases rapidly, then gradually decreases and stabilizes over time. Similar results regarding the variation of water temperatures in buried pipes during the summer were reported in references [34] and [37], indicating the reliability of the findings in this paper.

Furthermore, previous studies primarily focused on the heat exchange performance of GHEs or individual components of heat pump units. In contrast, this paper integrates the heat pump unit with the GHE. The objective is to examine how the heat exchange performance of the GHE and the unit’s cooling and heating capacity are affected when the unit reaches the protection temperature.

#### 4.1.2 Operation of two GHE.

**Fig 9:**
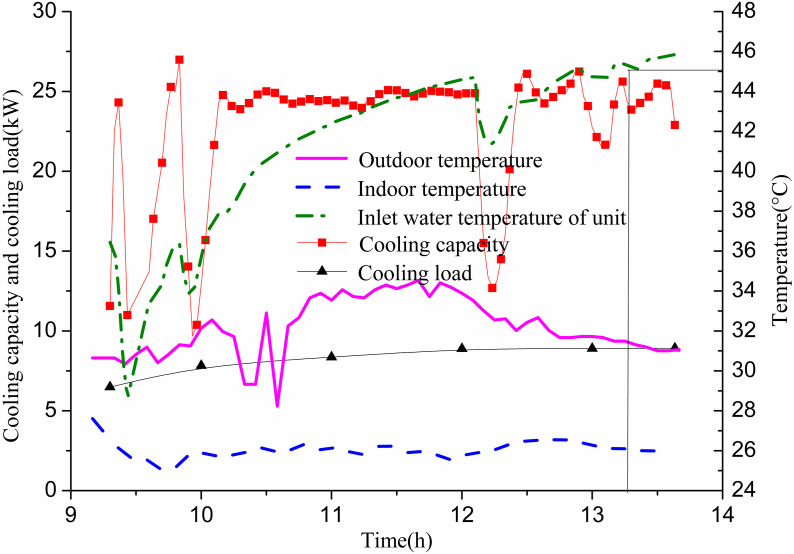
Variations of cooling capacity and indoor cooling load with operation time of two GHEs operating in summer.

**Fig 10:**
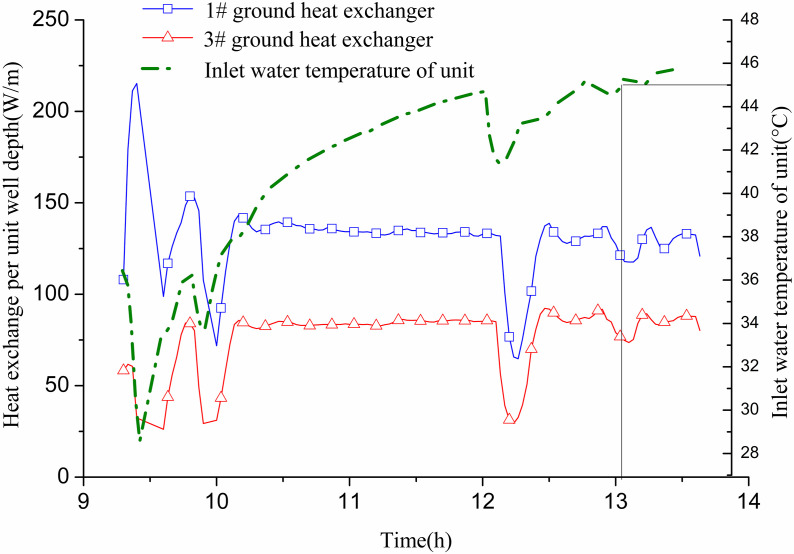
Variations of heat exchange per unit well depth and inlet and outlet water temperature with operation time of two GHEs operating.

On July 22, GHEs 1# and 3# were operational from 9:20 to 13:40. The flow rate for 1# was 0.5 m/s, while 3# had a flow rate of 0.48 m/s. Fig 9 illustrates the relationship between cooling capacity and indoor cooling load. The data shows that with both GHEs operating, the inlet water temperature of the heat pump unit rose from 28.6 °C to 45 °C in 3.93 h (3 h and 56 min). The cooling capacity consistently met the indoor cooling load, allowing the indoor temperature to reach the design target of 26 °C. According to Fig 10, the heat exchange per unit well depth for 1# is 119.8 W/m, while for 3#, it is 86.8 W/m. No well wall temperature measuring points were provided for the two GHEs, which precluded further analysis.

This section compares the performance of one GHE against two GHEs. During operation, the outdoor temperature ranged from 27–34 °C. The heat pump unit’s cooling capacity met indoor cooling demand effectively when both GHEs were in operation. With two GHEs available, heat exchange with the soil was sufficient. The water inlet temperature for the heat pump did not exceed the set limit, resulting in high operational efficiency. Consequently, the cooling supply adequately addressed the cooling load, and the indoor temperature reached the desired level.

### 4.2 Winter conditions

#### 4.2.1 One GHE operates separately.

##### (1) Experimental study:

**Fig 11:**
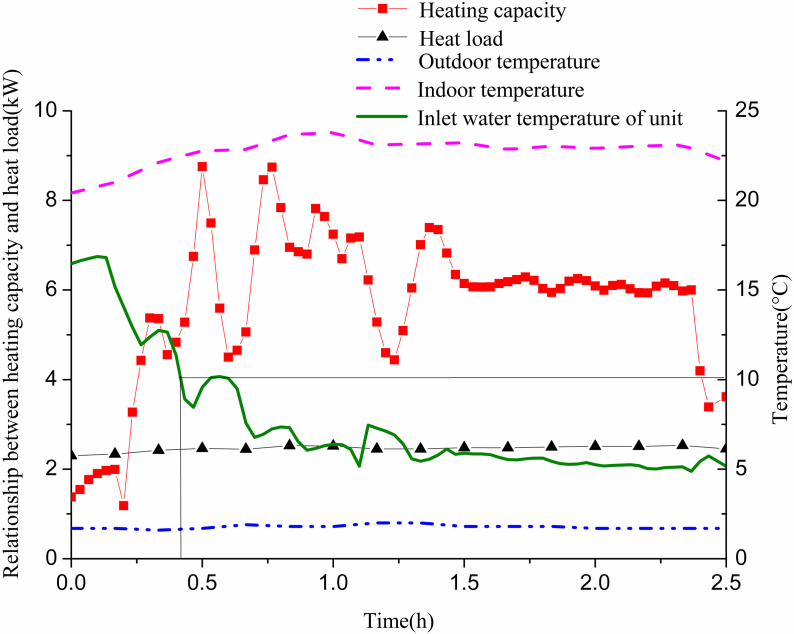
Variations of cooling capacity and indoor cooling load with operation time of single GHE operating in winter.

On January 5, from 19:30 to 22:00, GHE 12# was operated at a flow rate of 0.61 m/s. Fig 11 illustrates the system’s heating capacity in relation to indoor heat load during winter. The inlet water temperature of the GSHP unit dropped from 16.5° C to 10 °C in approximately 24 min (0.4 h) and further decreased to 5 °C in about 2.2 h. Initially, the heat supply could not meet indoor heat load demands within the first 15 min. This was due to a high inlet temperature and a small temperature difference between the water and soil, resulting in insufficient heat transfer. To meet indoor load requirements, the outlet temperature of the unit decreased, which subsequently reduced the inlet temperature of the GHE and led to a continuous decline in the unit’s inlet temperature. After this initial period, heat supply stabilized and adequately met the indoor load after 1.5 h. The indoor temperature was set to 22 °C, and the actual room temperature gradually increased from an initial 20.4 °C to a stable level of approximately 23 °C.

**Fig 12:**
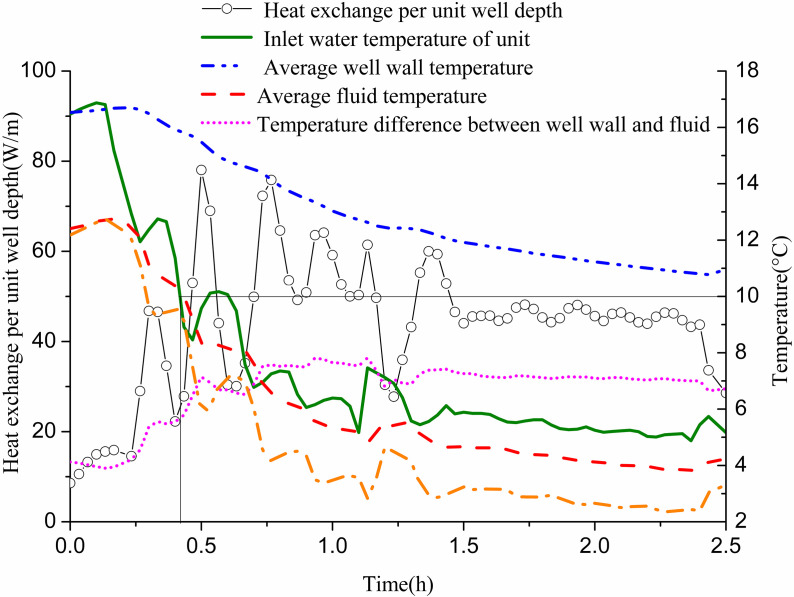
Variations of heat exchange per unit well depth and temperature difference between pipe wall and fluid with operation time of single GHE operating in winter.

Fig 12 shows the variation of heat exchange per unit well depth, along with the well wall and fluid temperatures over time. The heat exchange per unit well depth fluctuates significantly in the first 1.5 h and stabilizes during the following hour. Initially, due to the high inlet temperature of the GHE and the minimal temperature difference with the surrounding soil, heat exchange is low. As time progresses, the well wall temperature and fluid temperature gradually decrease. Initially, the temperature difference between them is small; however, it gradually increases and approaches stability. This behavior occurs as the inlet water temperature of the GHE decreases from approximately 12 °C to 3 °C to meet indoor heat demands. When the unit’s inlet water temperature reaches 10 °C, the temperature difference between the inlet and outlet of the buried pipe is 1.78 °C. At this point, the heat exchange per unit well depth measures 27.85 W/m. The well wall temperature is 15.82 °C, while the fluid temperature stands at 10.03 °C, resulting in a temperature difference of 5.79 °C. When the inlet water temperature drops to 5 °C, the temperature difference between the GHE’s inlet and outlet increases to 2.83 °C. The heat exchange per unit well depth then rises to 43.95 W/m. The well wall temperature is recorded at 11 °C, with the fluid temperature at 3.97 °C, yielding a temperature difference of 7.02 °C.

In Shanghai, winter heating demands are relatively low compared to summer cooling needs. Thus, operating a single GHE in winter allows the heat pump unit’s heating capacity to meet heat load requirements; however, the water inlet temperature may fall below the set limit, resulting in low heating efficiency. In areas with high heat load requirements, insufficient GHEs can lead to inadequate heat supply to meet demand.

##### (2) Comparison between theoretical calculation and experimental results:

**Fig 13:**
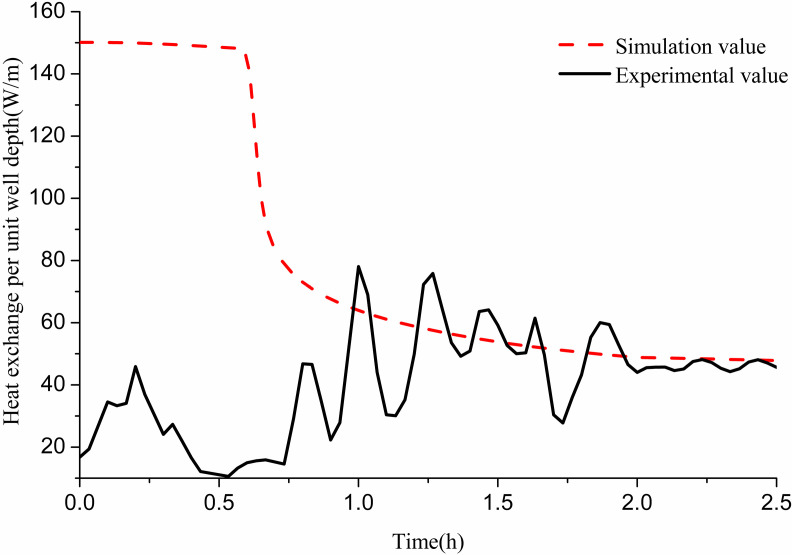
Comparison between experimental and simulated values of heat transfer per unit well depth of 12# GHE.

In the above experiment, the GHE operates for 2.5 h and recovers for 6 h. Numerical calculations are conducted under these experimental conditions. The parameters are as follows: a flow rate of 0.61 m/s, an average inlet water temperature of 7.7 °C, and an initial soil temperature of 17.3 °C at different well wall depths at the start of the experiment. Fig 13 presents the comparison between simulated and experimental values of heat exchange per unit well depth over time during the 2.5 h operation. The simulation results indicate that heat exchange is substantial initially, stabilizing and then gradually declining after 1 h. In actual operation, heat exchange fluctuates significantly with indoor load and stabilizes after 1.5 h. The average heat transfer per unit well depth after 1 h of actual operation is 50.09 W/m, while the simulation yields an average of 54.07 W/m, resulting in an error of 7.95% compared to experimental results.

**Fig 14:**
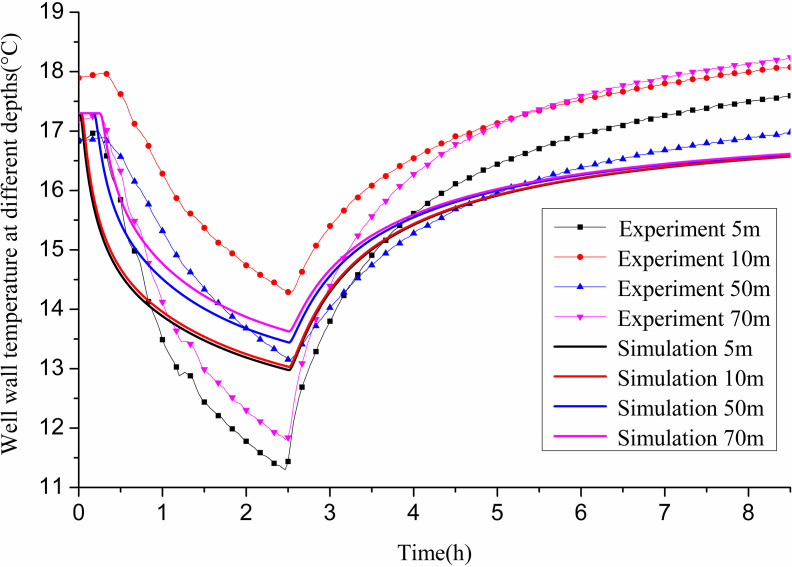
Comparison between experimental and simulated values of well wall temperature at different depths of 12# GHE.

Fig 14 compares the experimental and simulated values of well wall temperature at varying depths in the GHE. Both experimental and simulated results indicate a continuous increase in well wall temperature during operation, followed by gradual recovery after halting operation. At the end of operation, the simulated value at 5m depth is 12.98 °C, while the experimental value is 11.43 °C, resulting in an error of 13.56%. At 10m depth, the simulated value is 13.03 °C, and the experimental value is 14.29 °C, with an error of 8.81%. At 50m depth, the values are 13.44 °C (simulated) and 13.15 °C (experimental), with a 2.2% error. For 70m depth, the simulated value is 13.63 °C and the experimental value is 11.84 °C, yielding an error of 13.13%. During recovery, the simulated value at 5m depth is 16.57 °C, compared to 17.59 °C experimentally, resulting in a 5.8% error. At 10m depth, the simulated value is 16.58 °C versus 18.07 °C experimentally, yielding an 8.2% error. At 50m depth, the simulated value is 16.61 °C and the experimental value is 16.98 °C, with a 2.2% error. At 70 m depth, the simulated value is 18.24 °C while the experimental value is 16.62 °C, resulting in an 8.9% error. At the end of the operation, the errors between simulated and experimental values exceed 10% at 5m and 70m depths, while remaining below 10% for other depths.

#### 4.2.2 Operation of two GHE.

**Fig 15:**
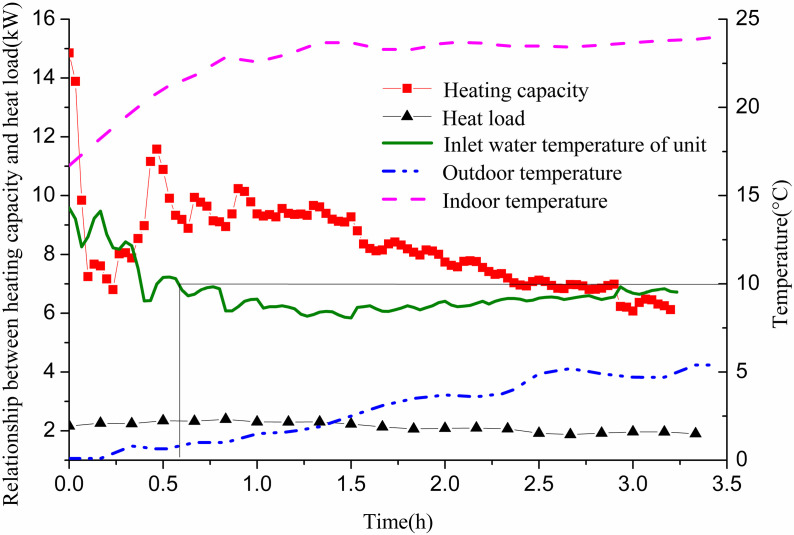
Variations of cooling capacity and indoor cooling load with operation time of two GHEs operating in winter.

From 9:00 to 12:20 on January 5, GHEs 11# and 12# were in operation. The flow rate for 11# was 0.25 m/s, while 12# had a flow rate of 0.58 m/s. Fig 15 illustrates the relationship between the system’s heating capacity and indoor heat load. It shows that the inlet water temperature of the GSHP unit decreased from 14.3 °C to 10 °C in approximately 36 min. The inlet water temperature remained below 10 °C for the next 3 h. Initially, the heating capacity was high with significant fluctuations, but it gradually trended downward while consistently meeting indoor heat load requirements. The indoor temperature was set to 22 °C, and the actual room temperature rose continuously from an initial 16.7 °C, eventually stabilizing around 23.5 °C.

**Fig 16:**
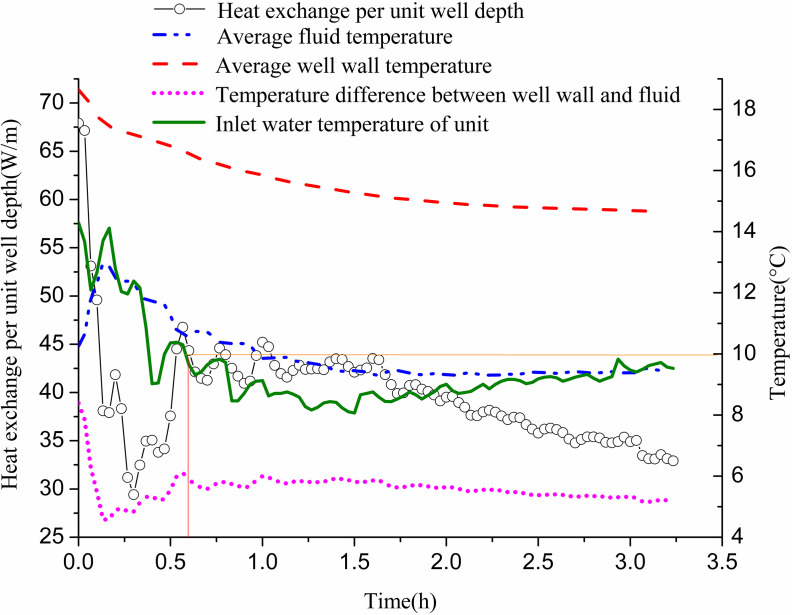
Variations of heat exchange per unit well depth and temperature difference between pipe wall and fluid with operation time of 11# GHE operating in winter.

**Fig 17:**
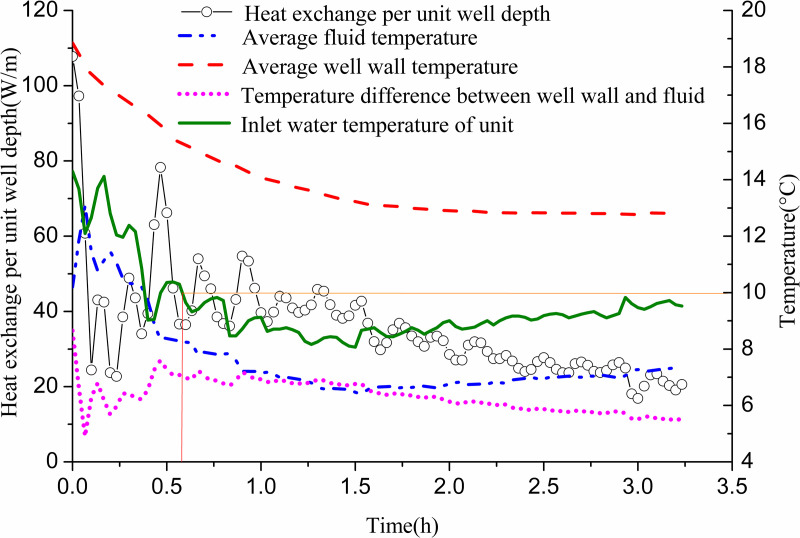
Variations of heat exchange per unit well depth and temperature difference between pipe wall and fluid with operation time of 12# GHE operating in winter.

Figs 16 and 17 display changes in heat exchange per unit well depth for GHEs 11# and 12# over time, along with well wall and fluid temperatures. Initially, heat exchange per unit well depth was high, exhibiting considerable fluctuation. This fluctuation decreased over time, as did the overall exchange rate. The well wall temperature gradually decreased, while the fluid temperature increased during the first 10 min before declining. Initially, the temperature difference between the well wall and fluid was substantial, but this difference gradually reduced. When the inlet water temperature of the unit reached 10 °C, the temperature difference between the inlet and outlet water of GHE 11# was 7 °C. The heat exchange per unit well depth was 44.35 W/m, with an average fluid temperature of 10.64 °C and an average well wall temperature of 16.55 °C, creating a temperature difference of 5.9 °C. For GHE 12#, the inlet and outlet water temperature difference was 2.45 °C, with a heat exchange per unit well depth of 36.48 W/m. The average fluid temperature was 8.25 °C, and the average well wall temperature was 15.22 °C, resulting in a temperature difference of 6.98 °C. At the end of the operation, GHE 11# showed a temperature difference of 5.17 °C between inlet and outlet water, with a heat exchange per unit well depth of 32.9 W/m. The fluid temperature was 9.49 °C, and the well wall temperature was 14.68 °C, leading to a temperature difference of 5.18 °C. For GHE 12#, the temperature difference between the inlet and outlet water was 1.38 °C, with a heat exchange per unit well depth of 20.58 W/m, a fluid temperature of 7.32 °C, and a well wall temperature of 12.83 °C, resulting in a temperature difference of 5.51 °C.

### 4.3 Overall heat transfer coefficient

The overall heat transfer coefficients of a GHE operating under different conditions during summer and winter are calculated separately. In summer, the coefficients were calculated at three stages: when the inlet temperature of the heat pump unit reached 45 °C after 2.67 h of operation, when the cooling supply matched the cooling load after 0.5 h of continuous operation, and when the cooling supply was 90.1% of the cooling load at the end of the experiment. In winter, the coefficients were calculated when the inlet temperature of the heat pump unit dropped to 10 °C after 0.4 h of operation and when it decreased further to 5 °C after 2.2 h of operation. The calculation results are shown in Table 5.

**Table 5:** Comprehensive heat transfer coefficient.

Season	Stage	*Q* (W)	*q’* (W/m^2^)	*t*_b_ (°C)	*t*_f_ (°C)	*t*_b_*-t*_f_ (°C)	K (W/(m^2^ °C))
summer	The inlet temperature of the heat pump unit reached 45 °C	10774.53	329.9	36.14	40.31	4.17	79.12
	The cooling supply was equal to the cooling load	10537.57	322.68	34.04	37.35	3.31	97.49
	The cooling supply was 90.1% of the cooling load	9412.68	288.24	36.98	39.71	2.73	105.58
winter	The inlet temperature of the heat pump unit decreased to 10 °C	2228	68.23	15.82	10.03	5.79	11.78
	The inlet temperature of the heat pump unit decreased to 5 °C	3516	107.67	10.99	3.97	7.02	15.34

As seen in Table 3, during summer, heat transfer between the GHE and the soil causes the soil temperature to rise, which in turn raises the water inlet temperature of the GHE. This results in a decrease in the temperature difference between the well wall and the fluid, leading to reduced heat transfer. However, the reduction in heat transfer is less than the reduction in the temperature difference, which causes the heat transfer coefficient to increase with continued operation. In winter, as heat is exchanged between the GHE and the soil, the soil temperature decreases, which lowers the water inlet temperature of the GHE. This increases the temperature difference between the well wall and the fluid, enhancing heat exchange and raising the heat transfer coefficient.

## 5 Conclusion

(1) In this experiment, one GHE was evaluated during summer. When the inlet water temperature of the heat pump unit reached 45 °C, the cooling capacity adequately met the cooling load demand, with a heat exchange rate of 134.4 W/m per unit well depth. At the point when the cooling capacity just met the cooling load demand, the heat exchange rate dropped to 131.5 W/m. With continued operation, the cooling supply failed to meet the cooling load, resulting in a heat exchange rate of 118.47 W/m. When two GHEs operated, it took 3.93 h for the inlet water temperature of the unit to reach 45 °C. The heat exchange rates for the two exchangers were 119.8 W/m and 86.8 W/m, respectively. This indicates that when the number of GHEs is insufficient in summer, the exchangers cannot effectively transfer heat with the soil. Consequently, the inlet temperature of the heat pump unit increases, leading to a decrease in the unit’s refrigeration efficiency, and the cooling capacity fails to meet the cooling load requirements.
(2) Regarding winter operation with one GHE, when the inlet water temperature of the heat pump unit reached 10 °C, the heat exchange per unit well depth was 27.85 W/m. When the inlet temperature dropped to 5 °C, the heat exchange rate increased to 43.95 W/m, which allowed the heat supply to meet indoor heating load demands. With two GHEs operational, the inlet water temperature at 10 °C yielded heat exchange rates of 44.35 W/m and 36.48 W/m for the two units, respectively. Although the heat supply met the demand when running one GHE in winter, the inlet temperature of the heat pump unit fell below the set limit. This drop resulted in reduced heating efficiency and decreased heat production.

## 6 Limitations and prospects

This study examined heat exchange per unit well depth, the temperature difference between the fluid and the soil, and the inlet temperature of the heat pump unit when the cooling and heating capacity could not meet load demands. It was demonstrated that insufficient buried pipes or extended operation time could hinder the cooling and heating capacity of the heat pump unit from fulfilling indoor demands. While this research provides valuable insights for engineering applications, it also has limitations.

(1) The experiment took place in Shanghai, focusing on small-scale buildings. The data collected was from a specific day, making it non-representative of all climate zones or larger structures.
(2) The sample size of the experimental data is too limited to offer meaningful quantitative insights for engineering practice, providing only qualitative analysis.
(3) In the actual GSHP system with buried pipes, the pipes interact with each other. However, this experiment involved only one or two pipes, which may not reflect the complexities of a full buried pipe group operation.

Future research directions include:

(1) Developing a model of the buried pipe GSHP system using numerical simulation software. This would help explore the operational limits of buried pipes in various regions and conditions, providing a quantitative basis for engineering applications.
(2) Investigating the limits of the heat pump, as well as the inlet and outlet temperatures of the GHE and the achievable ground temperature over extended periods of buried pipe operation.

## Supporting information

S1 File List of experimental and simulation data under different working conditions. (XLSX)
